# Injection Molding and Sintering of ZrO_2_ Ceramic Powder Modified by a Zirconate Coupling Agent

**DOI:** 10.3390/ma15197014

**Published:** 2022-10-10

**Authors:** Qiangyi Mao, Liang Qiao, Jingwu Zheng, Yao Ying, Jing Yu, Wangchang Li, Shenglei Che, Wei Cai

**Affiliations:** 1 College of Materials Science and Engineering, Zhejiang University of Technology, Hangzhou 310014, China; 2 Research Center of Magnetic and Electronic Materials, Zhejiang University of Technology, Hangzhou 310014, China

**Keywords:** injection molding, ZrO_2_, zirconate coupling agent, sintering

## Abstract

Ceramic injection molding is a near-net shape-processing technique, producing ceramic components with low tooling costs and complex shapes. In this paper, ZrO_2_ ceramics with high loading content in the green part were prepared by powder modification using zirconate coupling agent, injection molding and sintering, which benefited decreasing the usage of binders and deformation of ceramics. The rheological characteristics of feedstocks, densities, microstructures and mechanical properties of green and sintered parts with the different coupling media and sintering temperatures were studied. The results showed that the addition of a zirconate coupling agent with ethanol medium obviously increased the flowability of feedstocks and benefited achieving the green parts with high powder loading (86.5 wt.%) and bending strength (12.9 MPa) and the final unbroken ceramics. In addition, the sintering temperatures from 1500–1575 °C had no significant effects on the density, hardness, and surface morphology of the ceramic samples. However, the bending strength increased and some large grains with transgranular fracture occurred on the fractural surface at the sintering temperature of 1575 °C.

## 1. Introduction

Zirconia ceramics have become one of the widely concerned materials in the field of 5G communication and smart wearable devices due to high strength, no EMI (Electromagnetic Shielding), low sensitivity, strong corrosion resistance, wear resistance, and other comprehensive properties [1,2,3,4]. At the same time, the good biocompatibility also makes it have good application prospects in the field of medical repair [5,6,7]. However, due to the high hardness and fragility of zirconia ceramics, the traditional machining is difficult and often causes fragmentation and damage [8,9].

Ceramic injection molding (CIM) is a combination of powder technology and injection molding [10,11,12,13]. As a near-net shape-processing technique, CIM can produce ceramic components with low tooling costs and complex shapes [14,15]. In the past years, the study of ceramic injection molding mainly focused on the formulation of binder systems and debinding. Hanemann et al. [16] designed an environmentally friendly water soluble binder system contains polymethacrylate and polyethyleneglycol for ZrO_2_ ceramic injection molding. Delaroa et al. [17] found that a thinner wax network was promoted with the increasing HDPE content in the framework adhesive system, which limited the diffusion distance and favored an increased ceramic densification. Wen et al. [18] studied the effects of different backbone binders in the molding, debinding, and sintering process and found that the multi-polymer (LDPE and HDPE) backbone binder showed the most suitable characteristics for injection molding. Ye et al. [19] found that microcrystalline wax in the binder system decreased the viscosity of feedstock and increased the solvent debinding ratio of the green part in the injection molding of Sr-ferrite. Using LDPE, HDPE, and wax binders, Zhao et al. [20] achieved complete ZrO_2_ ceramics injection molding with a powder loading of 85.5 wt.%. Liu et al. [21] found that the debinding temperature was the main factor affecting the debinding rate in the early stage of solvent debinding. Oliveira et al. [22] investigated the influence of specimen geometry and temperature on the solvent debinding of Al_2_O_3_ molding specimens and found that the solvent debinding rate increased with the increase of surface-to-volume ratio and debinding temperature. Trunec et al. [23] and Tseng et al. [24] revealed that the cracks and defects became obvious when the heating rate increased to a critical level (30 °C/h) during single thermal debinding in Al_2_O_3_ and ZrO_2_ ceramic injection molding. Ani et al. [25] also found that combining suitable solvent debinding and thermal debinding process could successfully remove all binders from the injection parts and avoid the formation of defects in the ATZ ceramic injection molding.

Because surfactants and coupling agents can effectively improve the compatibility, dispersion and combination between ceramic powders and polymer matrix in the resin-based composites [26,27,28], some researchers used them in ceramic injection molding. Liu et al. [29] found that stearic acid formed a uniform coating layer on the surface of zirconia powder, improving the rheology of feedstocks and the solvent debinding ratio. Hanemann et al. [30] also reported that stearic acid as surfactant effectively improved the filling volume of ceramic powders in LDPE and PW binders. Edirisinghe et al. [31] and Zhang et al. [32] used a silane coupling agent to improve the rheological behavior of the feedstocks and promote the debinding process of Si_3_N_4_ ceramic injection molding. Takahashi et al. [33] found that the coupling agents reduced the apparent viscosity of the zirconia feedstocks for injection molding in the order of titanate > silane > aluminate with 2 wt.% additions. Deng et al. [34] also found that the addition of a silane coupling agent decreased the shear viscosity of the feedstocks and increased the solvent binder removal ratio, which benefited the ZrO_2_ ceramic injection molding. Liu et al. [35] achieved the highest relative densities of the sintered parts at 1450 °C using a titanate coupling agent to modified ZrO_2_ powder, 100 °C lower than the unmodified powders.

In this study, we found that a zirconate coupling agent benefited increasing the ZrO_2_ powder loading due to the great flowability improvement in injection molding, which benefited decreasing the usage of binders, increasing the debinding efficiency and deformation of ceramics. The high content (86.5 wt.%) of ZrO_2_ powder loading was used and the rheological behavior, microstructure, and mechanical properties of ZrO_2_ ceramics by injection molding using a zirconate coupling agent for the powder modification were studied in detail.

## 2. Experimental

Commercially available yttria-stabilized zirconia powder (3Y-TZP) (CAS: 1314-23-4, 99% ZrO_2_(HfO_2_) + Y_2_O_3_, SZ-DM3.0-F2, Jiangxi size materials Co., Ltd., Jiujiang, China) with a primary particle size of about 80 nm was used as the raw material. A commercially available zirconate coupling agent (CAS: 22830-18-8, (C(CH_2_COOC_2_H_5_)_2_COOH)_2_Zr(OC_3_H_7_)_2_, Nanjing Pinning Coupling Agent Co., Ltd., Nanjing, China) with 0.75 wt.% content was used for surface modification of ZrO_2_ powder. The binder system consisted of high-density polyethylene (HDPE) (CAS: 25213-02-9, HDPE8050, Formosa Plastics Corporation, Taiwan, China), low-density polyethylene (LDPE) (CAS: 9002-88-4, LDPE2426K, Sinopec Maoming Petrochemical Company, Maoming, Guangdong, China), paraffin (PW) (CAS: 308069-08-1, China Petrochemical Corporation, Beijing, China), microcrystalline paraffin (MW) (CAS: 63231-60-7, China Petrochemical Corporation), stearic acid (SA) (CAS: 57-11-4, Shanghai Ling Feng Chemical Reagent Co., Ltd., Shanghai, China), and dibutyl phthalate (DBP) (CAS: 87-74-2, Sinopharm Chemical Reagent Co., Ltd., Shanghai, China).

**Figure 1:**
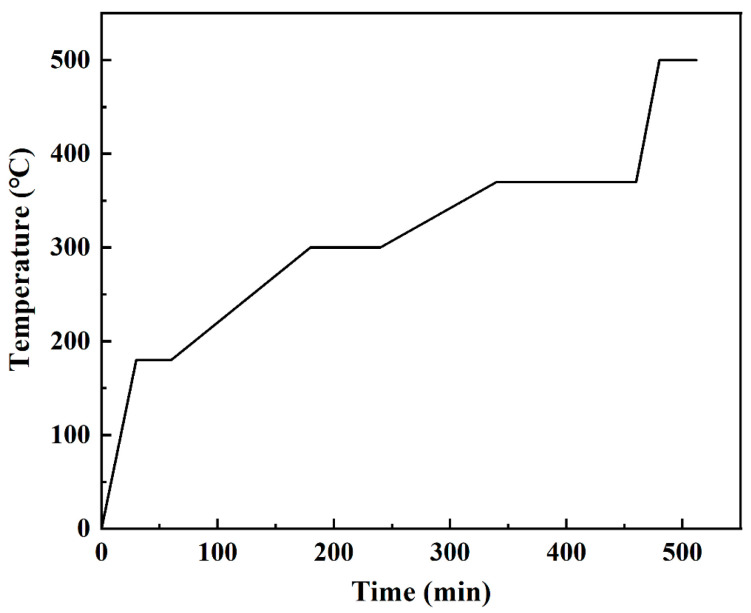
Thermal debinding process for the samples [34].

The surface modification process of ZrO_2_ powder was carried out in different coupling media containing ethanol and deionized water with different weight ratios as shown in Table 1. The modified ZrO_2_ powders and the binders were thoroughly mixed by a twin-screw extruder (SHJ-20B) at 135 °C with a screw speed of 18 rpm to obtain the peanut particles. The particles were then injected into rectangular green parts with the size of 80.00 mm × 10.00 mm × 4.00 mm by the injection molding machine (HTF60W2-Ⅱ) under an injection pressure of 70 MPa and temperature of 150 °C. Each sample contained at least five bars for the measurements. The two-stage debinding process containing solvent and thermal debinding was used to remove the binders in the green parts. First, the green parts were placed in n-heptane solvent at 45 °C for 8 h to remove the soluble binders (PW, MW, SA, DBP). Then, they were put into the muffle furnace to remove the backbone binders (HDPE and LDPE) with the controlling heating rate from room temperature to 500 °C according to the heating process as shown in Figure 1 based on our previous work [34]. After debinding, the samples were heated up to 1500–1575 °C at 5 °C/min and sintered for 2 h.

Rheological characteristics of feedstocks were measured by capillary rheometer (Rosand RH7) with the shear rates ranging from 10 to 10,000 s^−1^ at 150 °C. The melt index (MI) of feedstocks was measured using a melt flow instrument (XNR-400A) at 150 °C and 6200 g. The binder removal ratio of the green parts after solvent debinding was calculated by the mass change divided by the total mass of the soluble binders rate (PW, MW, DBP, and SA) when it was immersed in n-heptane at 45 °C for 8 h. The morphologies of the sintered samples were observed by scanning electron microscopy (SEM, HITACHI SU1510) and field emission scanning electron microscope (FE-SEM, HITACHI Regulus 8100). The particle size distribution was measured using Image J. The densities of sintered samples were measured by the Archimedes method. The Dual Column Tabletop Testing Machine (Instron 5966) was used to measure the bending strength of the green and sintered parts with the standard of ISO 6872: 2008. The Vickers hardness (HV) of the sintered samples was measured using a microhardness tester (HV-1000) in a load of 2.94 N and the dwell time was 10 s with ISO standard of ISO 14705-2000.

## 3. Results and Discussion

### 3.1. Effect of Coupling Media

In order to determine the influence of water as coupling media, the zirconate coupling agent was dropped directly into different coupling media with different weight contents of ethanol and deionized water. The coupling agent could be completely dissolved in ethanol and form clear solution. However, white precipitates formed immediately when the weight ratio of deionized water increased to more than 2%, indicating that the coupling agent was prone to hydrolysis and formed precipitates. Differently, the coupling agent directly floated on the solution surface in the form of oil droplet due to lipophilicity and finally also became white precipitates when the weight ratio of deionized water increased to 60%. The appearance of white precipitate revealed the inhomogeneous distribution of coupling agent and the decline of surface modification effect.

**Figure 2:**
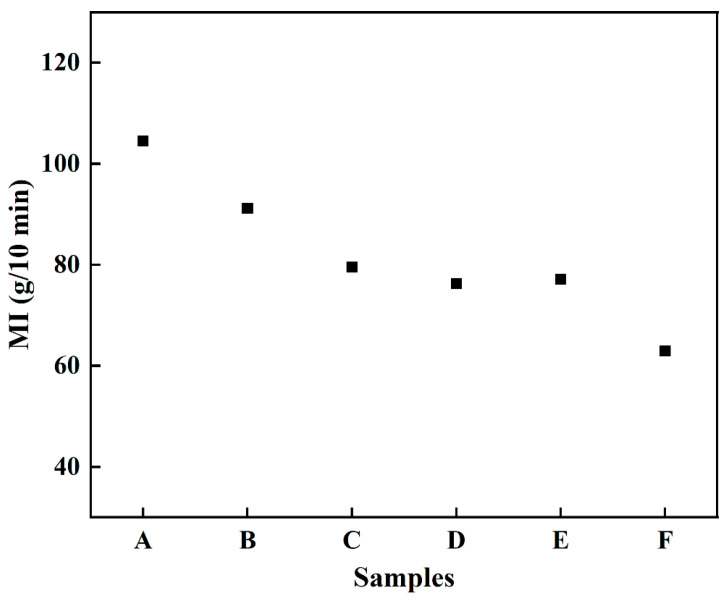
Melt index of sample A–F with different coupling media.

**Figure 3:**
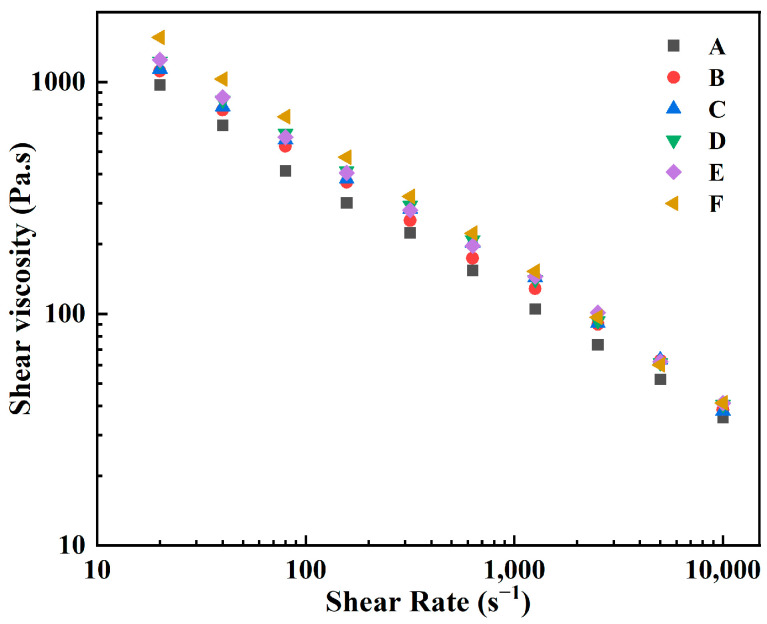
Rheological characteristics of samples A–F with different coupling media.

Figure 2 shows the melt index results of feedstocks with different coupling media. It could be seen that the MI results of feedstocks with surface modification of the zirconate coupling agent (samples A–E) greatly increased compared with that without coupling agent (sample F). In addition, the MI decreased with the increase of deionized water in the coupling media. The highest MI using ethanol as coupling medium reached 104.5 g/10 min (sample A). Figure 3 shows the shear viscosity of the feedstocks (samples A–F) with shear rates. The feedstocks displayed a similar rheological behavior, and the shear viscosity decreased with the increase of the shear rate, which agreed with the characteristic of pseudoplastic fluid. However, the shear viscosities of the modified samples were lower than that without coupling agent. In addition, the shear viscosity increased with the increase of deionized water in the coupling medium, consistent with the MI results as shown in Figure 2. Since the coupling agent was prone to hydrolysis and form precipitates when more than 2% water existed, using anhydrous ethanol as coupling media promoted the dispersibility of ZrO_2_ powder in the binders and greatly improved the flowability of feedstocks. On the contrary, the presence of deionized water in the coupling system reduced the modification effect. MI decreased and shear viscosity increased with the increase of water contents in coupling media.

**Figure 4:**
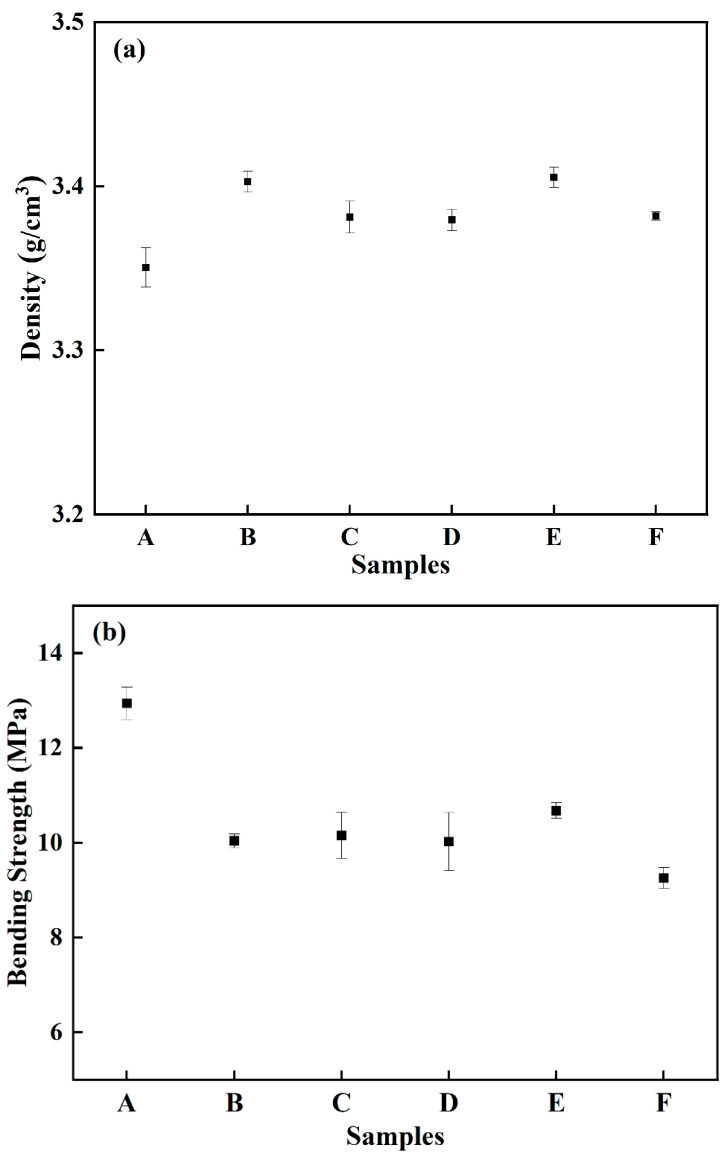
Densities (**a**) and bending strengths (**b**) of green parts with different coupling media.

Figure 4 shows the densities and bending strengths of the green parts with different coupling media. It can be seen in Figure 4a that the change of coupling media had no obvious effect on the densities of the green parts. The densities were between 3.35 and 3.41 g/cm^3^ with little difference. However, the bending strengths of modified samples (A–E) obviously increased compared with that without a coupling agent (sample F) as shown in Figure 4b. As the increase of deionized water in the coupling medium, the bending strengths of the green parts slightly decreased. The maximum strength reached 12.9 MPa when pure ethanol was used as the coupling medium (sample A).

**Figure 5:**
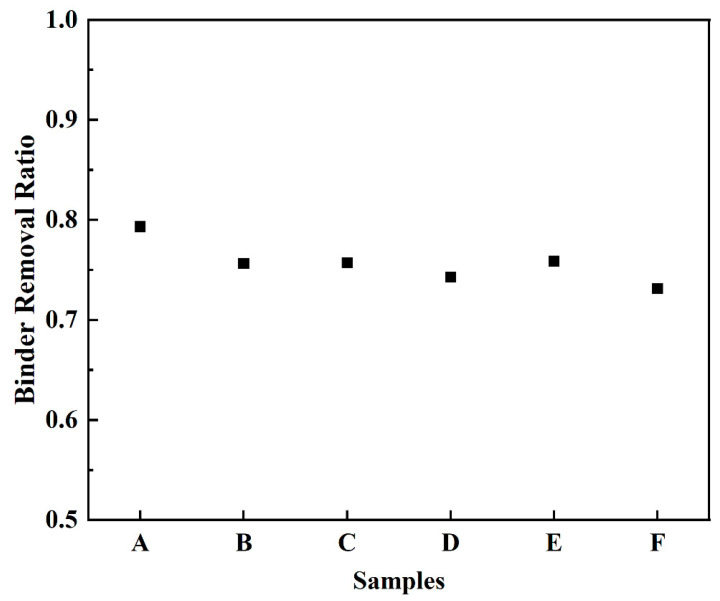
Binder removal ratios of green parts (sample A–F) after solvent debinding for 8 h.

The binder removal ratios of soluble binders during solvent debinding are shown in Figure 5. It could be seen that the removal ratio of sample A (79.3%) was higher than that of samples B–F (around 75%). The solvent debinding is a dissolution–diffusion process of the soluble binder in the solvent, which is strongly associated with the contact of binder and solvent. Since some soluble binders were covered with the backbone binders and separated from the solvent, the soluble binders could not be completely removed at this stage. The higher solvent debinding ratio in sample A indicated that the addition of coupling agent promoted the dispersibility of the soluble binder in the green parts due to the good flowability in the mixing and molding.

**Figure 6:**
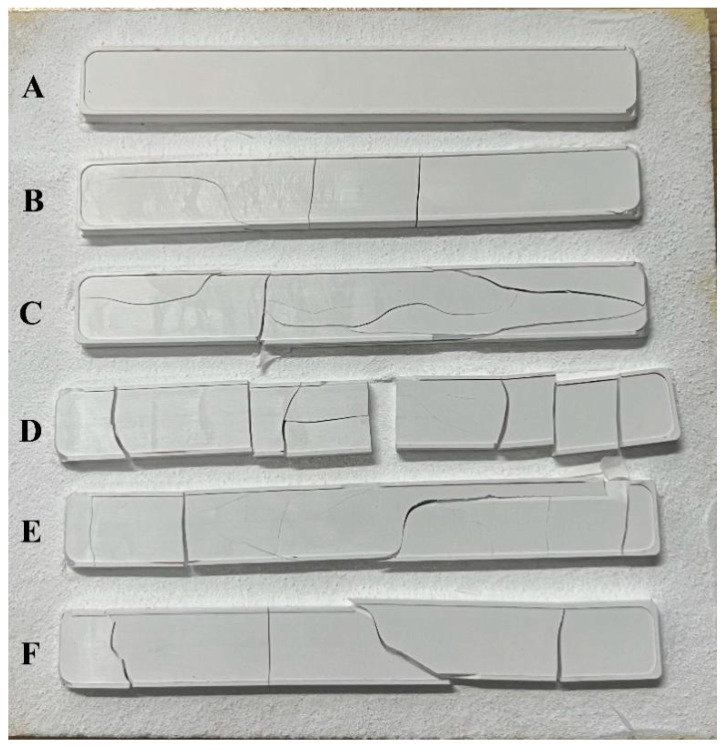
Photographs of green parts (samples A–F) after thermal debinding.

To observe the difference of samples A–F after thermal debinding, they were naturally cooled in the furnace from 500 °C to room temperature, instead of heating up to the sintering temperatures. As shown in Figure 6, all the green parts except sample A displayed various visible cracks in the surface and even broke after thermal debinding, which indicated that the coupling had a large influence on the crack occurrence of the samples after thermal debinding since the densities of green parts had little difference (Figure 4a). It is well known that the decomposition products of the backbone binders and remained soluble binders during thermal debinding are removed through the continuous pathway generated in the solvent debinding. Therefore, sufficient and uniform solvent debinding benefits the successful completion of thermal debinding. At the same time, if the backbone binders are not sufficiently distributed and gather together, the combustion products cannot be continuously eliminated in time and result in the formation of cracks. The sample A with high MI, rheological characteristics, solvent debinding ratio, and bending strength, associated with the good dispersion of the binder system in the green parts, ensured the gentle removal of the backbone binders and avoided forming cracks and defects during thermal debinding.

**Figure 7:**
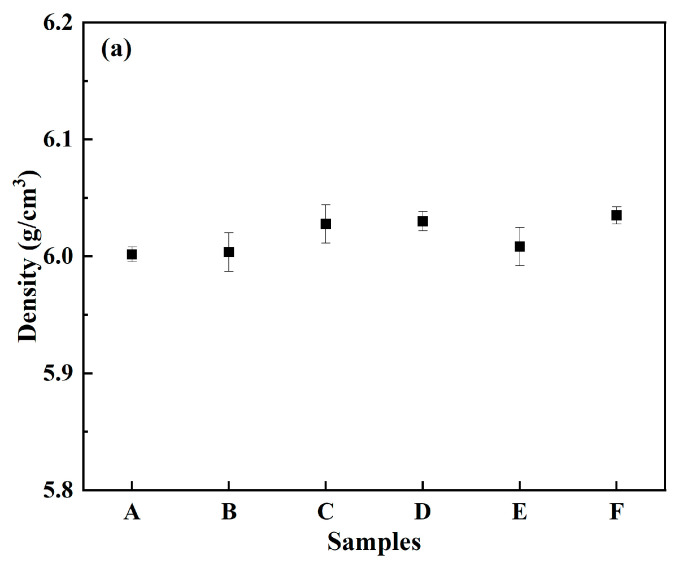

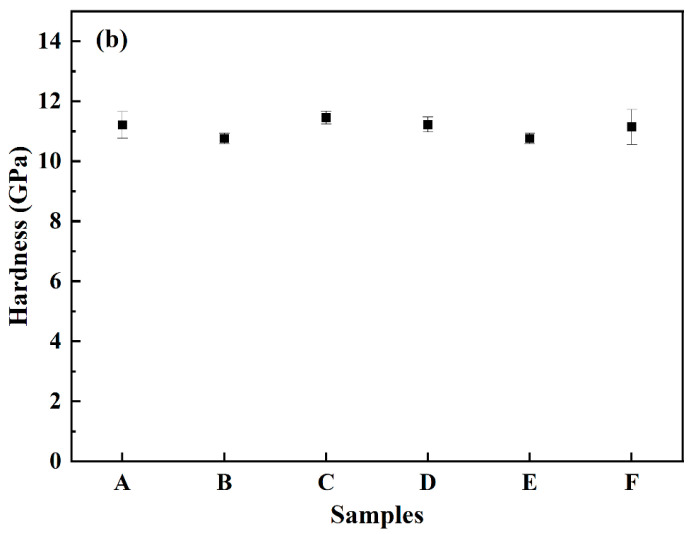
Densities (**a**) and hardness (**b**) of sintered samples with different coupling media at 1575 °C for 2 h.

Though the green parts of samples B–F were broken after thermal debinding, they were sintered at 1575 °C for 2 h with sample A for density and hardness measurements. As shown in Figure 7, the densities and hardness were between 6.00 and 6.03 g/cm^3^ and 11.5–12.5 GPa, respectively. All the green parts could be densified with no significant difference in density and hardness.

**Figure 8:**
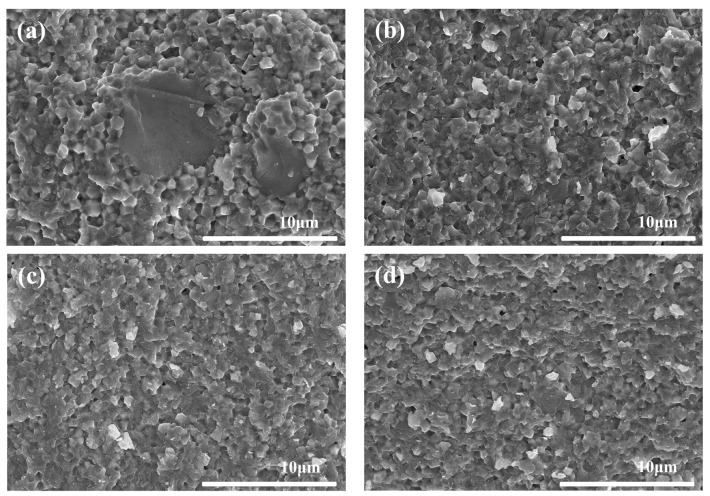

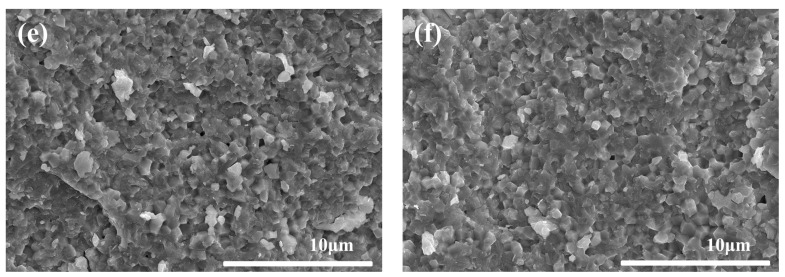
Fractural morphologies of the sintered samples with different coupling media at 1575 °C for 2 h. (**a**) sample A, (**b**) sample B, (**c**) sample C, (**d**) sample D, (**e**) sample E, and (**f**) sample F.

**Figure 9:**
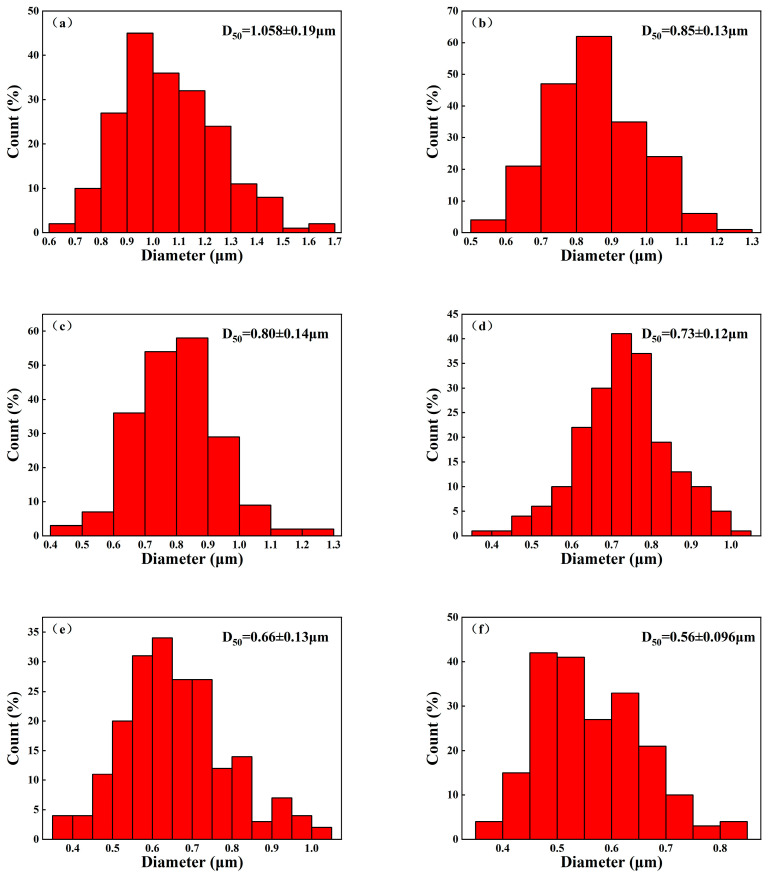
Particle size distribution of the sintered samples with different coupling media at 1575 °C for 2 h. (**a**) sample A, (**b**) sample B, (**c**) sample C, (**d**) sample D, (**e**) sample E, and (**f**) sample F. (Abnormally large grains were not involved in the statistics).

**Figure 10:**
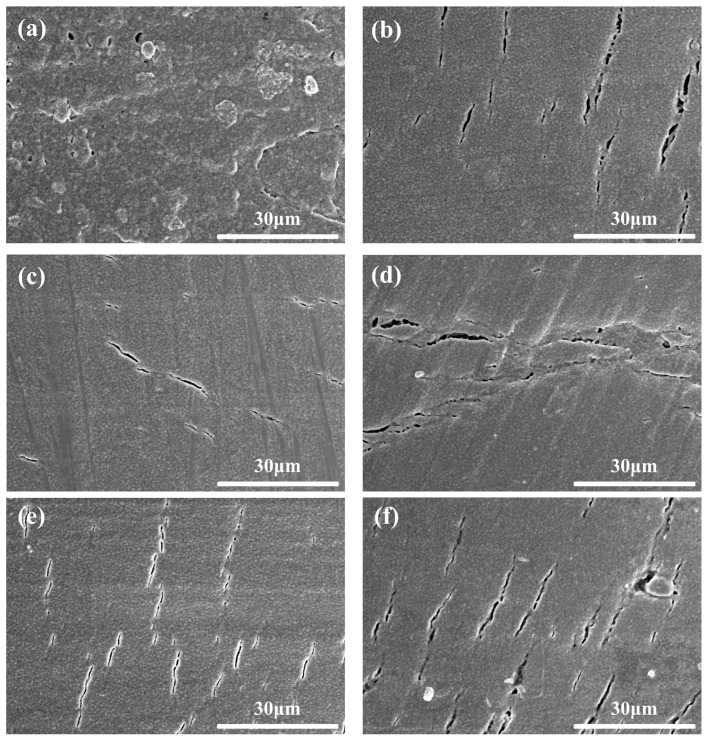
Surface morphologies of the sintered samples with different coupling media at 1575 °C for 2 h. (**a**) sample A, (**b**) sample B, (**c**) sample C, (**d**) sample D, (**e**) sample E, and (**f**) sample F.

Figure 8 shows the fractural morphologies of the samples with different coupling media at 1575 °C for 2 h. Figure 9 shows the particle size distribution of different samples in Figure 8. It could be seen that all the samples had a similar dense microstructure, consistent with the density and hardness results as shown in Figure 7. However, the grain size of sample A was a little larger than that of sample F as shown in Figure 8 and Figure 9. Meanwhile, it was strange to observe some large grains with the size of 3–10 μm in sample A, such as abnormal growth, which often occurs in ceramic sintering. As sample A was considered to have the best modified effect among all the samples, the zirconate coupling agent must promote the grain growth at 1575 °C. Figure 10 showed the surface morphologies of the sintered samples. It could be seen that sample A showed fewer and shorter surface cracks and defects compared with other samples. It also looked rougher because of the larger grains.

According to the Griffith Theory of brittle glass and ceramic materials, it was the existence of long cracks on the surface of samples B–F that resulted in the fracture of the green and sintered parts after thermal debinding. The long cracks forming on samples B–F could be impeded by the coupling modification of zirconia powder. As discussed in Figure 2, Figure 3, and Figure 4b, the zirconium propoxy group (Zr(OC_3_H_7_)_2_) in coupling agent hydrolyzed and bound to the hydrophilic zirconia powder surface, which improved the distribution between powders and binders and increased the flowability of feedstock. In addition, it was also found that the zirconate coupling agents could enhance the bonding between dental zirconia ceramics and resin composites, which provided better mechanical properties [36,37]. This benefited producing the homogeneous green parts with high bending strength. As shown in Figure 5, sample A had a higher solvent debinding ratio, which also decreased the formation and development of crack in the following thermal debinding. When the modification was missing or insufficient, the long crack easily occurred and even resulted in the fracture of the parts.

### 3.2. Effect of Sintering Temperature

**Figure 11:**
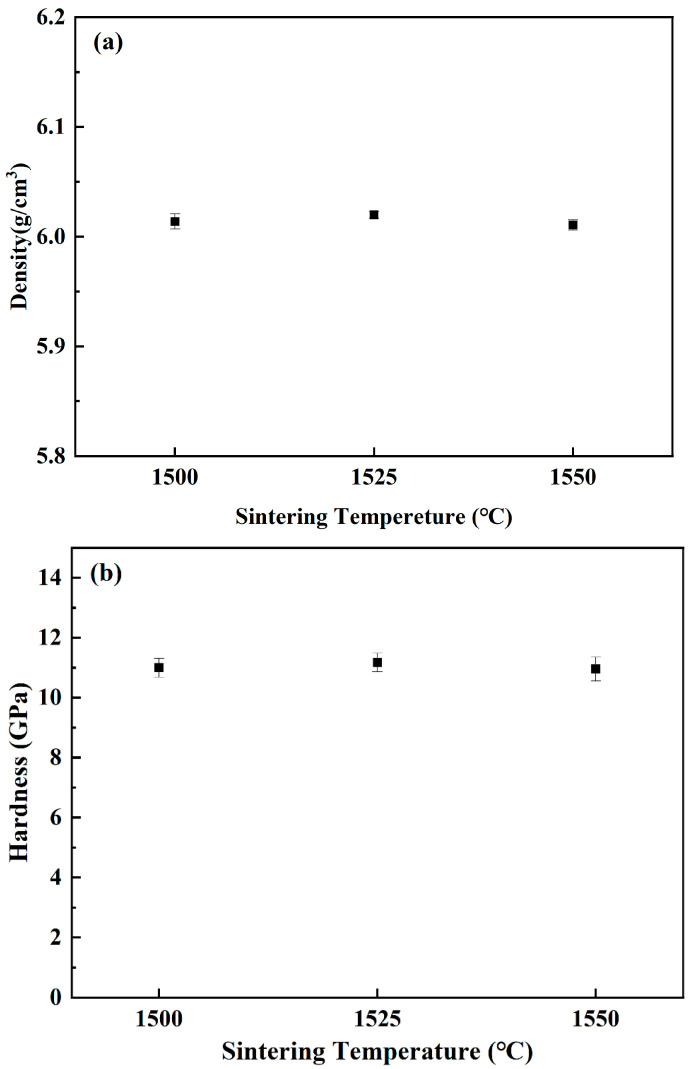
Densities (**a**) and hardness (**b**) of sample A sintered at different temperatures for 2 h.

Based on the abovementioned experiments, sample A was used to investigate the influence of sintering temperature. Figure 11 shows the densities and hardness of sintered parts at different sintering temperatures. Combined with Figure 7, when the sintering temperature increased from 1500 °C to 1575 °C, the density and hardness of sintered parts had no significant change, which were 6.00–6.02 g/cm^3^ and 10.5–11.5 GPa, respectively.

**Figure 12:**
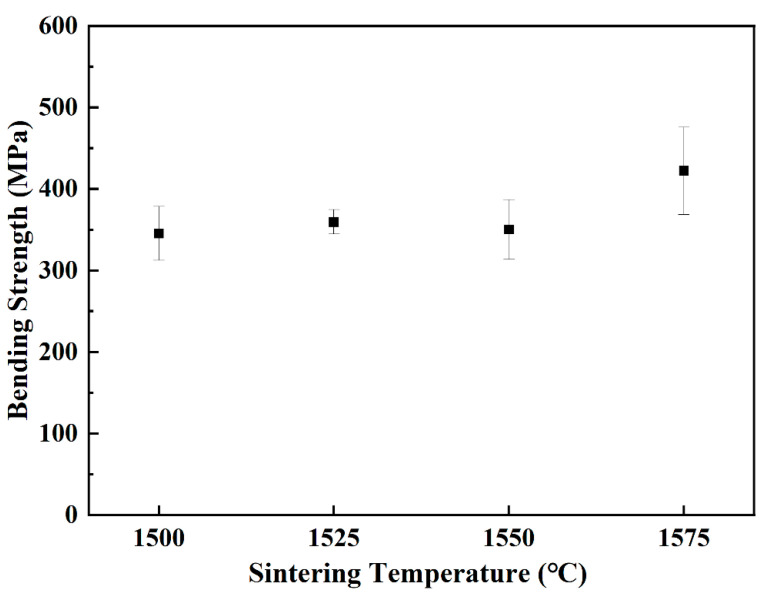
Bending strengths of sample A sintered at different temperature for 2 h.

Figure 12 shows the bending strengths of sintered parts with different sintering temperatures. It could be seen that the bending strengths were around 350 MPa when the sintering temperatures were between 1500–1550 °C. Differently, when the sintering temperature increased to 1575 °C, the bending strength increased and the average and maximum value of 422 MPa and 486 MPa were achieved respectively.

**Figure 13:**
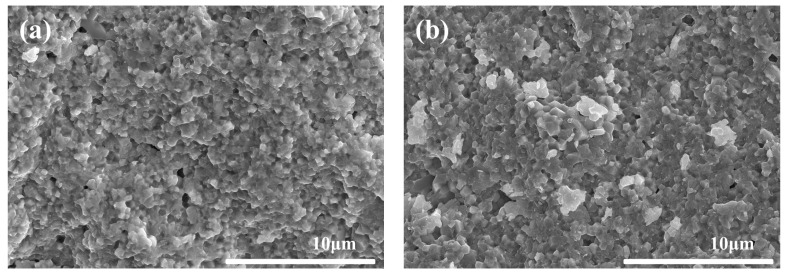

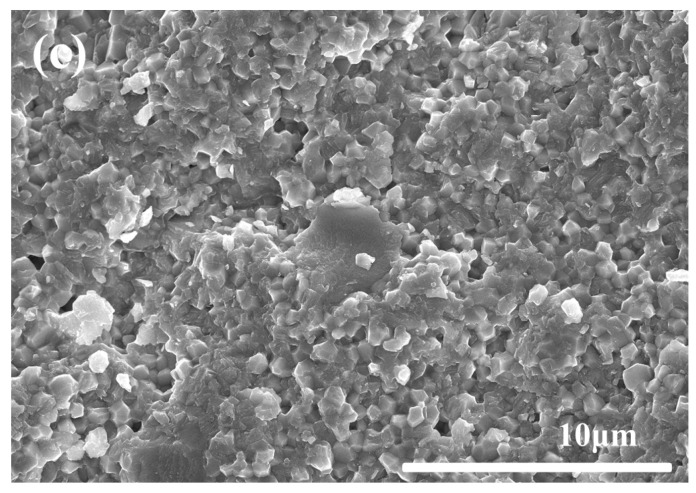
Fractural morphologies of the sintered parts at different sintering temperatures for 2 h. (**a**) 1500 °C, (**b**) 1525 °C, and (**c**) 1550 °C.

**Figure 14:**
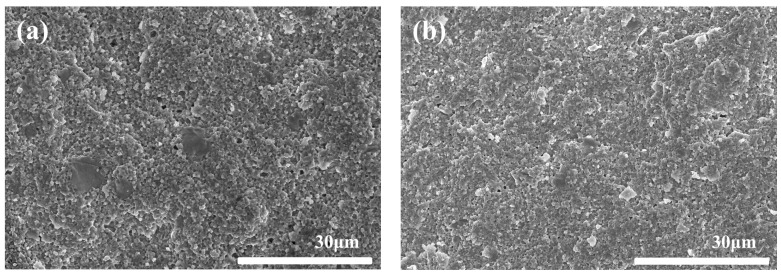
Comparison of fractural morphologies of the sintered parts at smaller magnification. (**a**) 1575 °C, and (**b**) 1550 °C.

**Figure 15:**
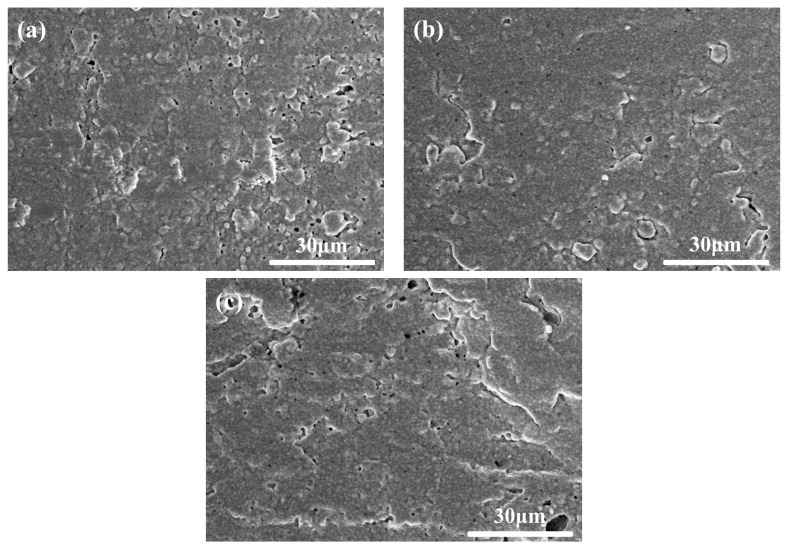
Surface morphologies of the sintered parts with different sintering temperatures for 2 h. (**a**) 1500 °C, (**b**) 1525 °C, and (**c**) 1550 °C.

Figure 13, Figure 14 and Figure 15 show the fractural and surface morphologies of the sintered parts at different temperatures for 2 h respectively. It was found that some large grains with size of 3–10 μm began to occur on the fractural surfaces when the sintering temperature increased to 1550 °C. Especially, when the sintering temperature was 1575 °C, their numbers and sizes increased compared with that at 1550 °C as shown in Figure 8a, Figure 13, and Figure 14. It could also be seen that the similar mini-pores and micro-cracks existed on the surface of sample A at different sintering temperatures from 1500–1575 °C combined with Figure 10a.

Since the density, hardness, and surface morphology of sample A had no significant difference at the sintering temperatures from 1500–1575 °C (Figure 10a, Figure 11, and Figure 15), the fractural morphology mainly influenced the bending strengths of sintered samples. Normally, the abnormal grain growth in the sintering would result in the decrease of ceramic mechanical properties. However, the large grains of 3–10 μm surrounded by the normal grains of about 1 μm just displayed transgranular fracture, different from intergranular fracture of the normal grains. Thus, the spreading of cracks along normal grain boundaries were suppressed and the strength was improved, as a dispersion-strengthening mechanism. Since the large grain was unobvious in samples B–F sintered at 1575 °C (Figure 8b–f) and sample A at 1500–1525 °C (Figure 13a,b), it may be associated with the joint action of high sintering temperature and homogeneous coupling modification. The detailed mechanism of growth needs further study in the future.

## 4. Conclusions

Using a zirconate coupling agent, the melt index increased and shear viscosity of the ZrO_2_ feedstocks decreased, which benefited injection molding. In the experiments, the maximum bending strength of the green parts was found to be 12.9 MPa at the high powder loading of 86.5 wt.%. The addition of deionized water in the coupling media had adverse effects on the modification. By comparison, the high MI, low shear viscosity, high bending strength, and high solvent binder removal ratio were achieved using pure ethanol as medium. In addition, it was found that the density (6.00–6.03 g/cm^3^) and hardness (11.5–12.5 GPa) of the sintered samples at 1575 °C had little change with the different contents of deionized water in the media. However, the green parts were easier to break after thermal debinding with the increase of water contents. When the sintering temperature increased to 1575 °C, the bending strength increased, and at the same time some large grains with transgranular fracture surrounded by the normal grains were observed. The abnormal grain growth was associated with the joint action of high sintering temperature and homogeneous coupling modification. The detailed mechanism of growth needs further study in the future.

## Figures and Tables

**Table 1:** Compositions of the samples with different coupling media.

Sample	Coupling Media (wt.%) W_ethanol_/(W_deionized water_ + W_ethanol_)	ZrO_2_ wt.%	HDPE wt.%	LDPE wt.%	PW wt.%	MW wt.%	DBP wt.%	SA wt.%
A	100	86.5	2.9	2.8	4.5	1.1	1.2	1.0
B	98	86.5	2.9	2.8	4.5	1.1	1.2	1.0
C	95	86.5	2.9	2.8	4.5	1.1	1.2	1.0
D	90	86.5	2.9	2.8	4.5	1.1	1.2	1.0
E	40	86.5	2.9	2.8	4.5	1.1	1.2	1.0
F	Unmodified	86.5	2.9	2.8	4.5	1.1	1.2	1.0

## Data Availability

The data presented in this study are available on request from the corresponding author.
